# Morphology of the genital organs of male and female giant anteaters (*Myrmecophaga tridactyla*)

**DOI:** 10.7717/peerj.11945

**Published:** 2021-08-11

**Authors:** Lilja Fromme, Débora R. Yogui, Mario Henrique Alves, Arnaud L.J. Desbiez, Marion Langeheine, André Quagliatto, Ursula Siebert, Ralph Brehm

**Affiliations:** 1 Institute for Terrestrial and Aquatic Wildlife Research, University of Veterinary Medicine Hannover, Hannover, Germany; 2 Institute for Anatomy, University of Veterinary Medicine Hannover, Hannover, Germany; 3 Project Anteaters and Highways, Instituto de Conservação de Animais Silvestres (ICAS), Campo Grande, Brazil; 4 Nashville Zoo, Nashville, TN, United States of America; 5 Fundación Zoológica de Cali, Valle del Cauca, Colombia; 6 Royal Zoological Society of Scotland (RZSS), Edinburgh, United Kingdom; 7 Instituto de Pesquisas Ecológicas (IPÊ), São Paulo, Brazil; 8 Laboratório de Ensino e Pesquisa em Animais Silvestres (LAPAS), Universidade Federal de Uberlândia, Uberlândia, Brazil

**Keywords:** Xenarthra, Reproduction, Anatomy, Histology, Persisting Müllerian duct, Persisting Wolffian duct, Persisting paramesonephric duct, Persisting mesonephric duct, Conservation

## Abstract

**Background:**

The giant anteater belongs to the supraorder Xenarthra which occupies a systematically isolated position among placental mammals. The species is categorized as Vulnerable by the International Union for Conservation of Nature, and understanding its reproductive characteristics is critical for future conservation efforts.

**Methods:**

Gross and microscopic anatomy of the genital organs of 23 male and 21 female adult and young roadkill giant anteaters in Brazil were studied.

**Results:**

Male giant anteaters presented a short conical penis, intraabdominal testes, and prostate, vesicular and bulbourethral glands. A tubular remnant of the partially fused Müllerian ducts extended from the seminal colliculus through the prostate gland, continued cranially in the genital fold, bifurcated, and attached with one elongation each to the left and right epididymal corpus. The structure presented a total length of up to 10 cm and contained a yellowish liquid in its lumen. Histologically, the caudal section of this structure resembled the female vagina, the middle portion corresponded to the uterus, and the extensions showed characteristics of uterine tubes. In adult female giant anteaters, ovoid ovaries with occasional seminiferous cord-like structures were observed. The animals possessed a simple uterus, which was directly continuous with the vaginal canal. The caudal portion of the vagina had two lumina, separated by a longitudinal septum and opening into two apertures into the vaginal vestibule, cranial to the urethral opening. In the urethral and the lateral vestibular wall, glandular structures with characteristics of male prostate and bulbourethral glands, respectively, were found. The vestibule opened through a vertical vulvar cleft to the exterior. A pair of well-differentiated Wolffian ducts with a central lumen originated ventrally at the vaginal opening into the vestibule and passed in a cranial direction through the ventral vaginal and uterine wall. Each duct extended highly coiled along the ipsilateral uterine tube until the lateral pole of the ovaries where it merged with the rete ovarii.

**Discussion:**

The reproductive morphology of giant anteaters reveals characteristics shared with other Xenarthrans: intraabdominal testes, a simple uterus, and a double caudal vagina. The persistence of well-differentiated genital ducts of the opposite sex in both males and females, however, singles them out among other species. These structures are the results of an aberration during fetal sexual differentiation and possess secretory functions. The possibility of a pathological degeneration of these organs should be considered in reproductive medicine of the species.

**Conclusion:**

Knowledge of the unique reproductive characteristics of the giant anteater is essential for future reproductive management of the species. Additionally, further research on the peculiarities of the persisting genital duct structures might help to understand sexual differentiation in placental mammals in general.

## Introduction

The giant anteater (*Myrmecophaga tridactyla*) belongs to the supraorder Xenarthra, which comprises anteaters, sloths and armadillos, and represents one of the four major clades of placental mammals (Delsuc et al., 2002; Gardner, 2008). In literature, a systematically isolated position of Xenarthrans in relation to the other three mammalian clades is discussed (Novacek, 1992; Gaudin & Croft, 2015), and the extraordinary phylogenetic status of the extant xenarthran species is pointed out (Vizcaíno & Bargo, 2014; Superina & Loughry, 2015), formerly including 31 (Gardner, 2008), more recently elevated to 39 species (Abba et al., 2015; Miranda et al., 2017; Feijó et al., 2019).

The giant anteater is the largest member of the anteater family and inhabits diverse ecosystems in Central and South America (Gardner, 2008; Miranda, Bertassoni & Abba, 2014). Nevertheless, its population is severely threatened by habitat loss and fragmentation, road-kills, wildfires, and species-specific characteristics such as low reproductive rates (Aguiar & Fonseca, 2008; Superina, Miranda & Abba, 2010; Miranda, Bertassoni & Abba, 2014). At present, the species is categorized as Vulnerable by the International Union for Conservation of Nature (IUCN, 2020) and Vulnerable (VU A2c) by the National Red List Brazil (Portaria MMA No. 444/2014, 2014). Efforts to guarantee the survival of this iconic species have to focus on habitat protection and mitigation of road-kills, but attention should also be given to the preservation of a healthy, genetically diverse captive population of giant anteaters (Miranda, Bertassoni & Abba, 2014). For both, in field conservation actions and reproductive management of captive populations, knowledge of reproductive features of a species is essential (Holt et al., 2003; Wildt et al., 2010).

Currently, there are only few and sometimes contradictory reports on the reproductive morphology of Xenarthrans. Previous studies on the genital organs of the male giant anteater have been limited to a macroscopic description and a brief outline of the histology of the testis (Kaudern, 1914; Grassé, 1955; Bartmann, Beyer & Wißdorf, 1991). In females, only macroscopic characteristics of the genital organs have been mentioned so far (Grassé, 1955; Schauerte & Osmann, 2012). There is a more detailed macroscopic and microscopic study on the reproductive morphology of the most closely related southern tamandua (*Tamandua tetradactyla*; Rossi et al., 2011; Rossi et al., 2012), and some literature on male and female armadillos (Rapp, 1852; Owen, 1868; Newfang, 1947; Enders, 1960; Glover, 1963; Cardoso et al., 1985; Cetica, Aldana Marcos & Merani, 2005) and sloths (Kaudern, 1914; Wislocki, 1928; Barretto, Amorim & Falcão, 2013; Favoretto et al., 2015). Some peculiar reproductive features have been portrayed in Xenarthrans, such as intraabdominal testes in males (Kaudern, 1914; Rossi et al., 2012), a simple uterus, and absence of a cervix in female specimens (Cetica, Aldana Marcos & Merani, 2005; Rossi et al., 2011), polyovular follicles in female armadillos of the family Chlamyphoridae (Cetica, Aldana Marcos & Merani, 2005), and polyembryony with identical quadruplets in the nine-banded armadillo (*Dasypus novemcinctus*; McBee & Baker, 1982). These data demonstrate the unique character of Xenarthrans and emphasize the necessity to study the anatomy of each species in detail.

The present study aimed to collect basic data on the reproductive morphology of giant anteaters, thus providing fundamental knowledge for future conservation efforts of the species.

## Materials & Methods

### Specimen collection

Reproductive tracts from 23 male and 21 female giant anteaters were collected during necropsies performed on road-kill specimens at the Federal University of Uberlândia (UFU, Minas Gerais, Brazil) and in cooperation with the Anteaters & Highways Project (Mato Grosso do Sul, Brazil). The samples were collected under the license no 49266-1 (UFU) and no 53798-10 (Anteaters & Highways Project) obtained by the Biodiversity Authorization and Information System (SISBIO, Brazil). The genetic resource access was registered under SisGen Cadastro no ADD1BBD.

### Age determination

Giant anteaters do not possess teeth (McDonald, Vizcaíno & Bargo, 2008), which are frequently used for age estimation in other wild animal species (Morris, 1972). Age determination was therefore based on the bodyweight of the specimens. In literature, a bodyweight (BW) of 22–39 kg is indicated for adult giant anteaters (Aguilar & Superina, 2015). Considering that the weight of the giant anteater carcasses used in the present study was sometimes reduced by the loss of tissue or blood due to vehicle collision, the following specimens were included: 21 adult male animals ≥ 28 kg BW, two young males <6 kg BW, 19 adult female animals ≥ 24 kg BW and two young females <2 kg BW.

### Macroscopic and microscopic analyses

During necropsies, the reproductive tract was examined *in situ* and was subsequently removed as a whole. The genital organs were studied macroscopically and morphometric measurements were taken. Representative samples of all structures were fixed in 10% neutral buffered formalin. Additionally, fragments of the testes were fixed in Bouin’s solution. Samples were then processed for histological analyses following standard protocols: dehydration by increasing concentrations of ethanol, clearance in xylene, and embedding in paraffin wax. For light microscopic observation, 5 µm thick tissue sections were mounted on slides and stained with hematoxylin and eosin.

## Results

Morphological findings were basically the same in young and adult specimens. Therefore, the description will not be subdivided according to age. However, some differences between age classes will be pointed out in the text.

### Sexual dimorphism

**Figure 1:**
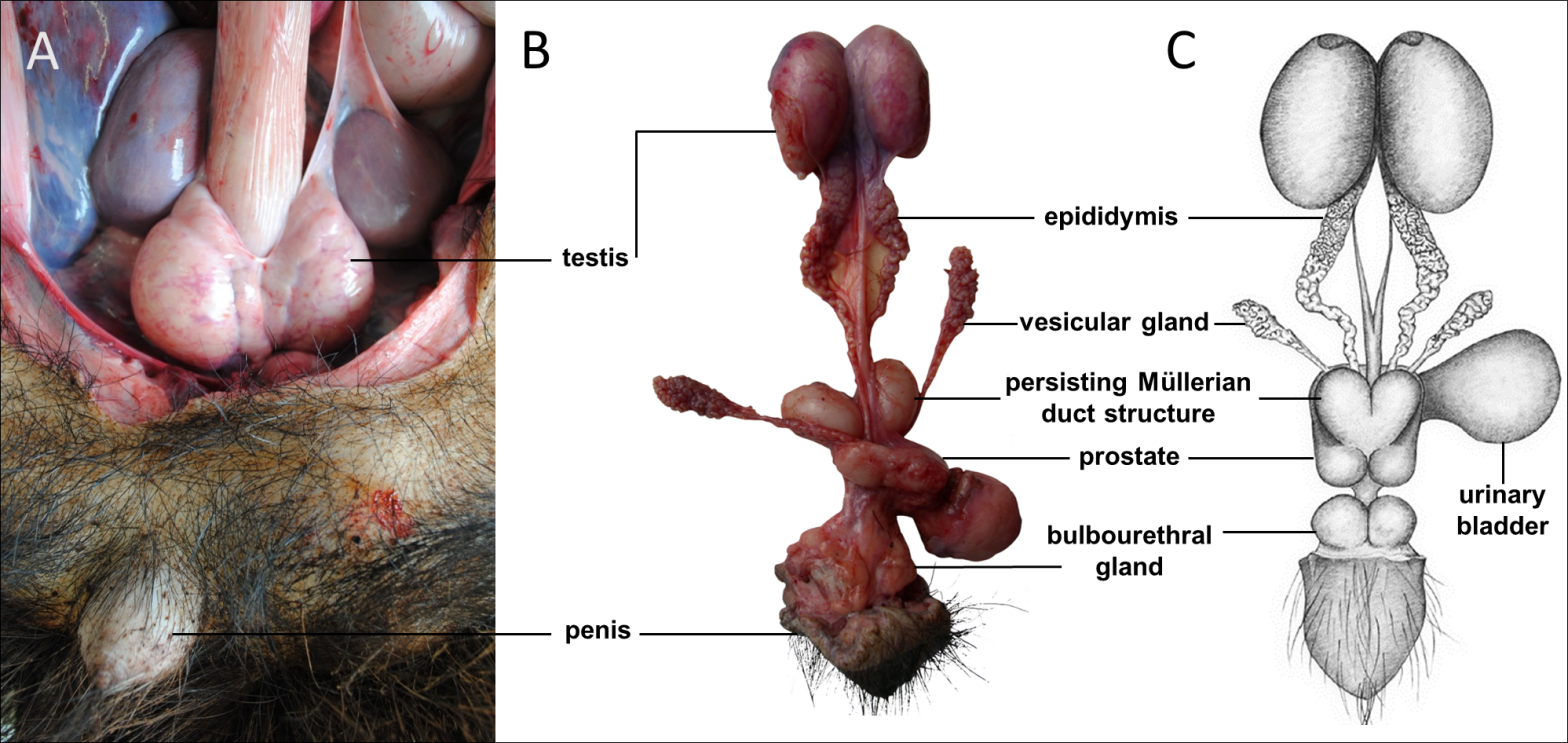
Macroscopic aspects of the genital organs of the adult male giant anteater. Genital organs (A) *in situ*; (B) dissected organs, ventral view; (C) schematic drawing of dissected organs, dorsal view.

Sexual dimorphism in giant anteaters was not apparent, and male and female animals could only be distinguished externally by close inspection of the external genital organs. As the species is testicond, no scrotum was visible in the male (Fig. 1), and even penis and vulva were similar in size and shape.

### Male genital organs

#### Testes, epididymides and deferent ducts

**Figure 2:**
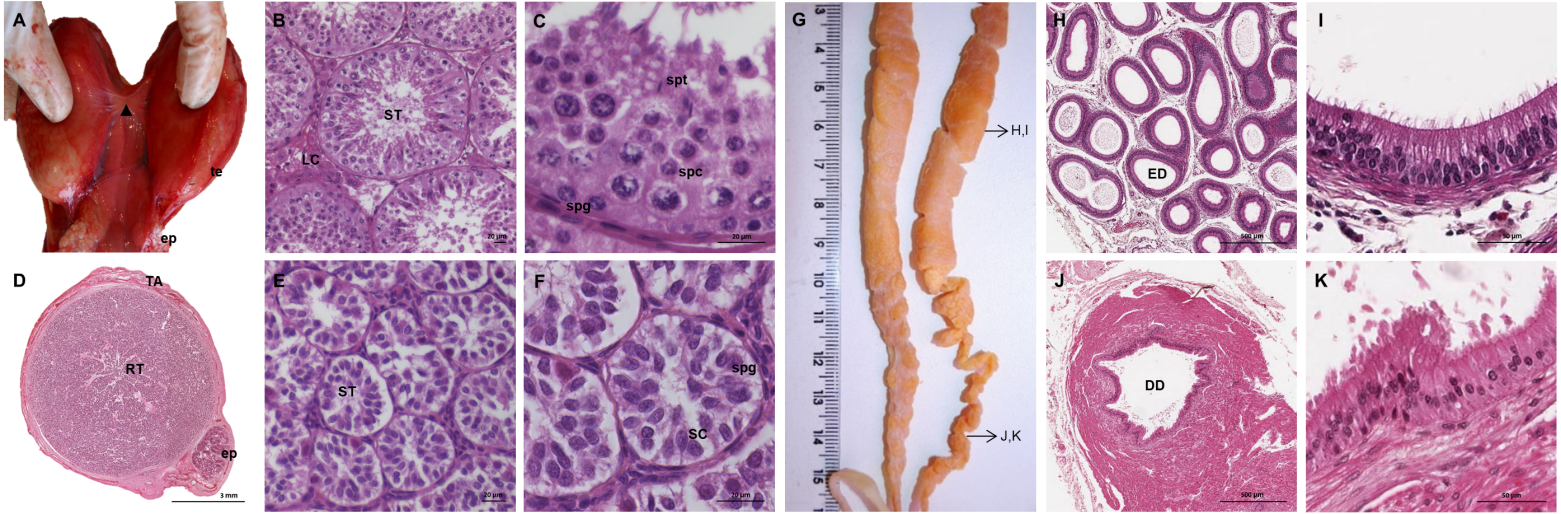
Macroscopic and microscopic aspects of the testes, epididymides and deferent ducts in the adult and young male giant anteater. (A–C) Testes of an adult giant anteater, (A) dorsal view of the testes, arrowhead: dense connective tissue, te: testis, ep: epididymis; (B) cross-section of a testis, ST: seminiferous tubules, LC: Leydig cells; (C) seminiferous epithelium in detail, spg: spermatogonia, spc: spermatocytes, spt: spermatids; (D–F) testis of a young giant anteater (D) cross-section of the testis, TA: tunica albuginea, RT: rete testis, ep: epididymis; (E) testis, ST: seminiferous tubules; (F) seminiferous tubules in detail, SC: Sertoli cell, spg: spermatogonia; (G) dorsal view of the epididymides and deferent ducts of an adult giant anteater, arrows indicating the sampled regions for histological sections; (H–I) cross-section of the epididymis, ED: epididymal duct; (J–K) cross-section of the deferent duct, DD: deferent duct. Hematoxylin-eosin staining.

The testes of the giant anteater were located in the caudal abdominal cavity, mediocaudally to the kidneys (Fig. 1A). They were oval, covered by a fibrous capsule, the tunica albuginea, and connected at the medial face by a strand of dense connective tissue (Figs. 1, 2A–2F). In adult specimens, the length of the right and left testes was 6–7 cm and the width and height measured 3–4 cm. Fine strands of connective tissue, eradiating from the tunica albuginea, divided the testicular parenchyma in lobules and merged centrally to form the mediastinum testis (Fig. 2D). The testicular parenchyma was composed of convoluted seminiferous tubules, and testicular stroma containing small clusters of Leydig cells and blood or lymphatic vessels (Figs. 2B, 2E). The seminiferous tubules were lined by a germinal epithelium, in which different stages of spermatogenic cells were observed in adult animals: spermatogonia, spermatocytes, spermatids, and spermatozoa in the tubular lumen (Fig. 2C). The ovoid nuclei of the Sertoli cells were located in the basal to medium compartment of the germinal epithelium. In young animals, the seminiferous tubules were composed only of Sertoli and some spermatogonial cells (Fig. 2F). The seminiferous tubules opened into the rete testis, a network of anastomosing tubules, located in the mediastinum and lined by a simple squamous epithelium. The rete testis emptied at the cranial testis pole *via* several efferent ductules into the epididymal duct, thus forming the head of the epididymis. The epididymal duct was lined by pseudostratified columnar epithelium with stereocilia, and surrounded by a layer of circularly oriented smooth muscle fibers. It was highly coiled and extended caudally in the epididymal body along the medial face of the testis (Figs. 2G–2I). Surpassing the caudal pole of the testis, the lumen of the epididymal duct and the smooth muscle layer increased gradually, continuing imperceptibly in the deferent duct, a thick-walled muscular tube with longitudinal mucosal folds and pseudostratified columnar epithelium (Figs. 2J, 2K). The deferent duct finally penetrated the prostate gland from dorsal and opened into the urethra on the seminal colliculus.

#### Accessory sexual glands

**Figure 3:**
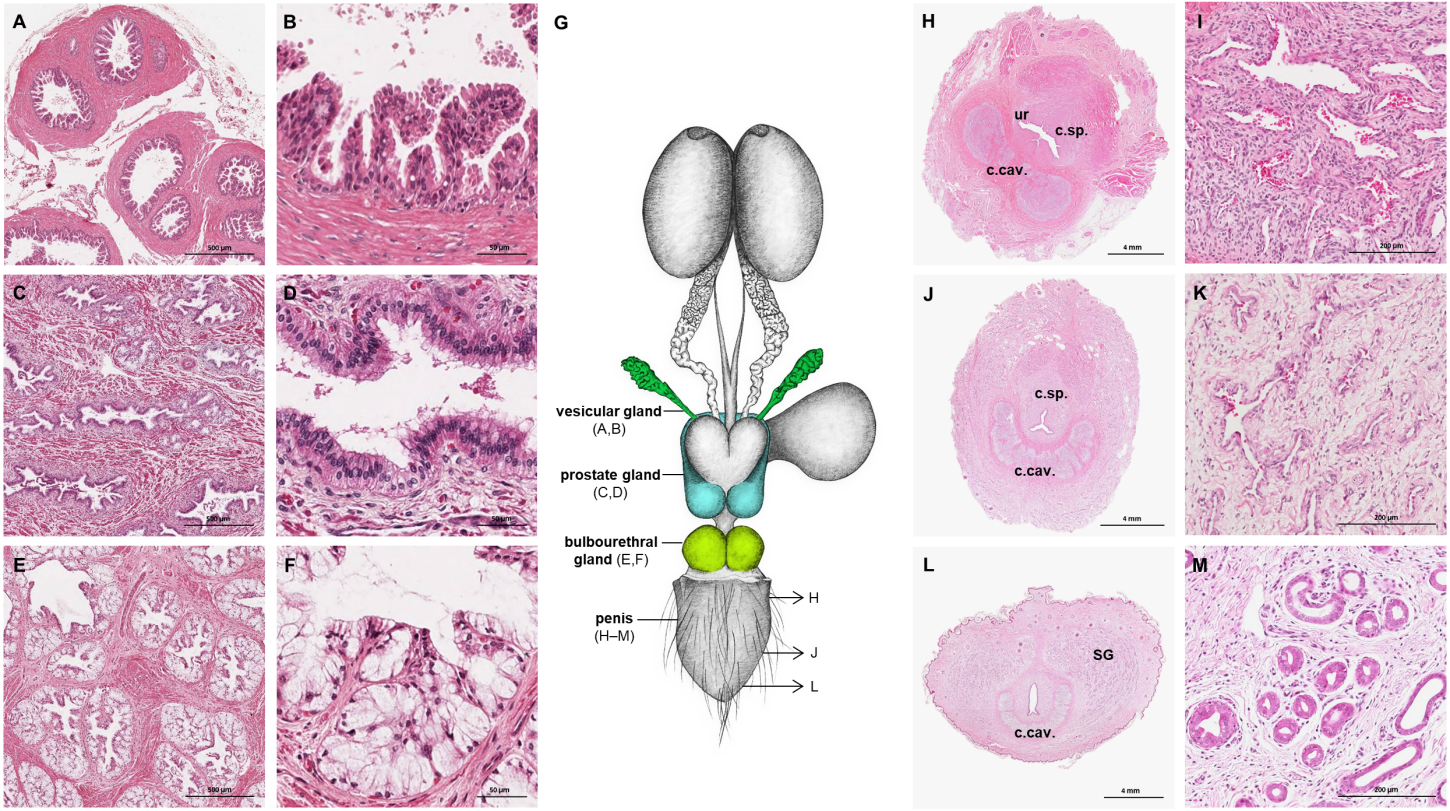
Macroscopic and microscopic aspects of the accessory sexual glands and the penis in the adult and young male giant anteater. (A–F) Accessory sexual glands in an adult giant anteater, (A–B) vesicular gland; (C–D) prostate gland; (E–F) bulbourethral gland; (G) schematic drawing of the genital organs, dorsal view, in color: accessory sexual glands, arrows: indicating the sampled regions for histological sections of the penis; (H, J, L) penis of a young giant anteater; (H) proximal cross-section, ur: urethra; c. sp.: corpus spongiosum, c.cav.: corpus cavernosum; (J) middle cross-section, c.sp.: corpus spongiosum, c.cav.: corpus cavernosum; (L) distal cross-section, c.cav.: corpus cavernosum, SG: sudoriferous glands; (I) corpus cavernosum; (K) corpus spongiosum; (M) sudoriferous glands. Hematoxylin-eosin staining.

The prostate gland was situated caudal to the urinary bladder (Fig. 1). It consisted of disseminated glandular tissue spread in the urethral wall and a compact glandular mass, englobing the urethra. The secretory units of the prostate gland, lined by pseudostratified epithelium, anastomosed with each other and drained into the urethra. The non-glandular stroma consisted of bundles of smooth muscle cells, separated by strands of dense connective tissue (Figs. 3C, 3D). The paired vesicular glands, elongated structures consisting of a coiled tube, were situated lateral to the terminal part of each deferent duct (Fig. 1). They penetrated the prostate gland and opened separately from the deferent ducts on the seminal colliculus. The mucosa formed numerous folds, lined by pseudostratified columnar epithelium, and was surrounded by a thick layer of smooth muscle (Figs. 3A, 3B). The distance from the free extremity of the penis to the seminal colliculus measured around nine cm in adult males. The bulbourethral glands were situated dorsolateral to the bulb of the penis (Fig. 1). They were ovoid-shaped and invested by a dense connective tissue capsule. The epithelium of the secretory units, mucous acini, was composed of simple columnar cells with abundant foamy cytoplasm and basal nuclei, and the excretory ducts opened into the urethra (Figs. 3E, 3F).

**Figure 4:**
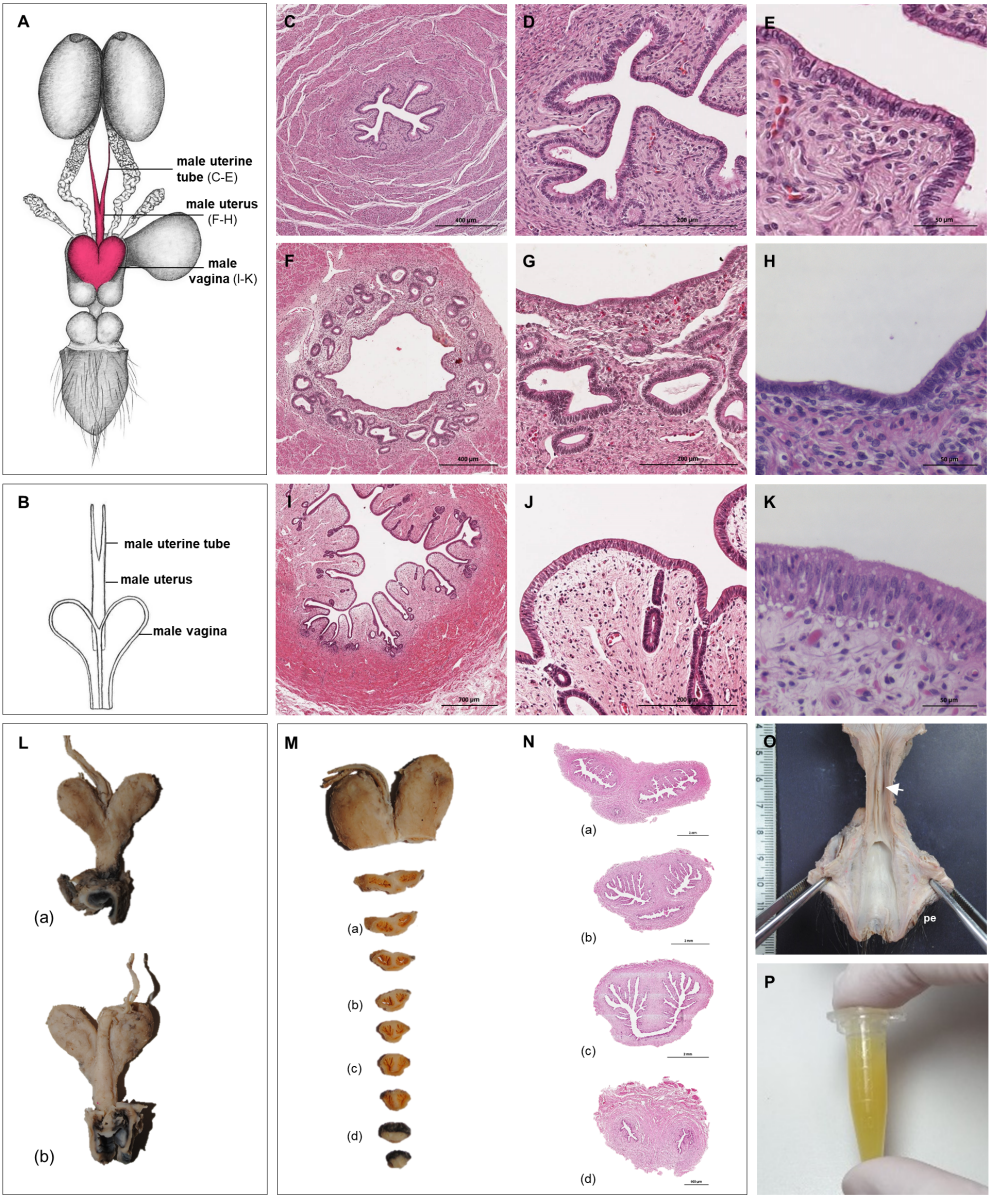
Macroscopic and microscopic aspects of the persisting Müllerian duct structures in the adult and young male giant anteater. (A) Schematic drawing of the genital organs, dorsal view, in red: persisting Müllerian duct structure; (B) schematic drawing of the persisting Müllerian duct structure, dorsal view, (C–K) cross-sections of the persisting Müllerian duct structure in an adult giant anteater; (C–E) distal segment with characteristics of a uterine tube; (F–H) middle segment with characteristics of a uterus; (I–K) proximal segment with characteristics of a vagina; (L) photographs of the dissected persisting Müllerian duct structure of a young giant anteater, (a): dorsal view, (b): ventral view and serial sections (M–N); (O) distal segment of the genital organs of a young male giant anteater, ventral view, longitudinally cut, arrow: seminal colliculus, pe: penis; (P) secretion found in the lumen of the persisting Müllerian duct structure. Hematoxylin-eosin staining.

#### Persisting Müllerian ducts

In all examined males, a tubular remnant of the partially fused Müllerian (or paramesonephric) ducts was observed, extending from the seminal colliculus to the epididymides (Figs. 1, 4). In its caudal portion, the muscular tube showed two separate lumina and passed from the seminal colliculus through the prostate gland, forming two vesicular dilatations dorsal to it. The mucosa of this section was thrown into numerous folds and lined by a columnar epithelium (Figs. 4I–4K). In some specimens, a murky yellowish fluid was observed in the lumen (Fig. 4P). After forming the vesicular dilatations, the two lumina fused into one lumen, and the tube continued cranially in the genital fold, medial to the deferent ducts. This part of the organ displayed a thick muscular wall, and a mucosa lined by a simple cuboidal epithelium with tubular glands opening on the epithelial surface (Figs. 4F–4H). After approximately 3–4 centimeters, the tubular structure bifurcated and attached with one elongation each onto the left and right epididymal corpus, at the medial face of the intraabdominal testes. In some males, these thin extensions showed a minute lumen and mucosal folds covered by a simple cuboidal epithelium (Figs. 4C–4E). In other specimens, a lumen was not detected. The Müllerian duct remnant had a total length of 7–10 cm in adult specimens, and showed a consistent morphology in all adult and young individuals. The microscopic features of the structure matched characteristics of the female genital organs: the caudal section with two lumina and two vesicular dilatations resembled the female vagina, the middle portion with a single lumen corresponded to a uterus, and both extensions showed characteristics of uterine tubes.

#### Penis

The penis was situated immediately ventral to the anus, resulting in a very short perineum. It was conical, was between 5 and 6 cm long in adult animals and directed caudally (Fig. 1). It was covered along its entire length by thick skin with occasional coarse hair. A prepuce was not formed, and the urethra opened through a small orifice on the free extremity. At the caudodorsal aspect of the penis, a median crest ran from the perineum to the urethral opening. The penis was composed of two erectile tissues: the paired ventral corpora cavernosa and the dorsal corpus spongiosum (Figs. 3H–3L). The corpora cavernosa were proximally divided by a strand of connective tissue, and fused distally to surround the corpus spongiosum in a semi-circle. The corpus spongiosum enclosed the urethra and narrowed distally, without forming a glans penis. Both erectile tissues consisted of cavernous spaces and trabeculae of connective tissue and smooth muscle fibers (Figs. 3I, 3K). In the distal penile segment, they were flanked by an agglomeration of numerous sudoriferous glands, lined by a simple cuboidal epithelium (Figs. 3L, 3M).

#### Female genital organs

### Ovaries

**Figure 5:**
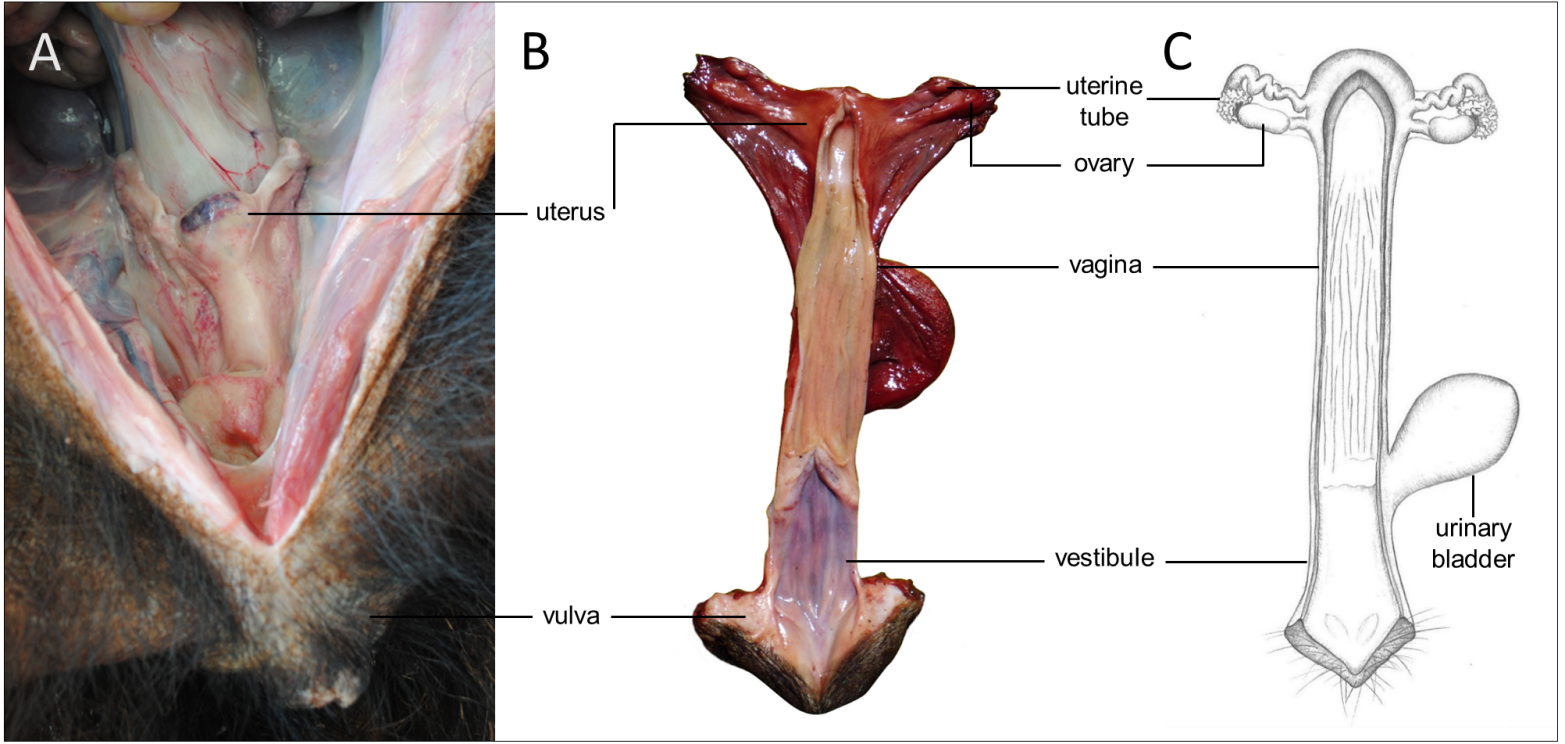
Macroscopic aspects of the genital organs of the adult female giant anteater. Genital organs (A) *in situ*; (B) dissected organs, dorsal view, longitudinally cut; (C) schematic drawing of dissected organs, dorsal view, longitudinally cut.

**Figure 6:**
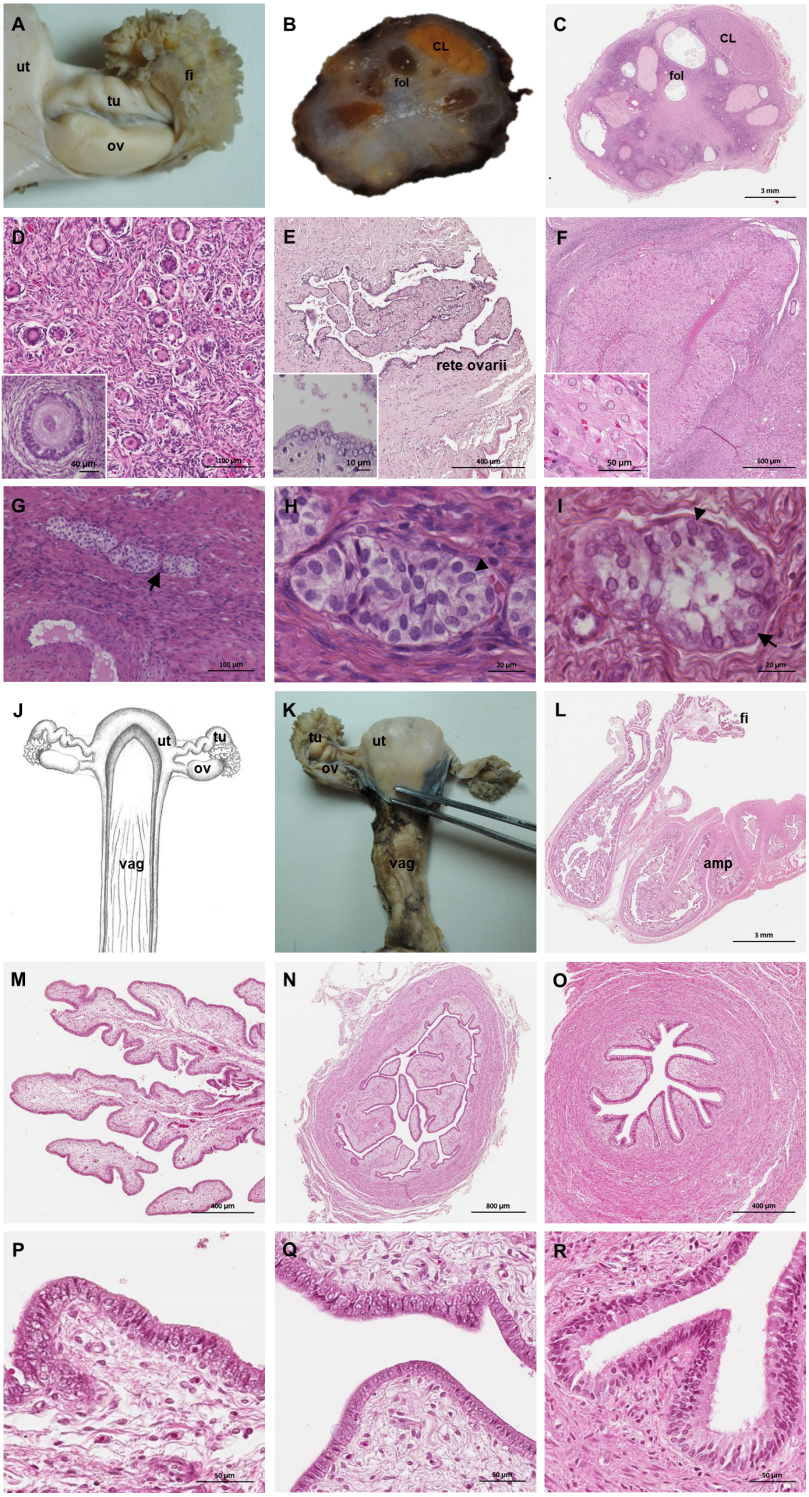
Macroscopic and microscopic aspects of the ovary and uterine tube of the adult female giant anteater. (A) Dorsal view of the ovary and uterine tube, ut: uterus, tu: uterine tube, fi: fimbriae, ov: ovary; (B) photograph and (C) photomicrograph of a longitudinal section through the ovary, CL: corpus luteum, fol: follicle; (D) ovarian tissue, inset: primary follicle; (E) lateral pole of the ovary with the rete ovarii, inset: epithelium of the rete ovarii; (F) corpus luteum, inset: luteal tissue; (G) ovarian tissue with cord-like structures (arrow); (H–I) cord-like structures in detail, arrowheads: ovoid nuclei, arrow: spherical nucleus; (J) schematic drawing and photomicrograph (K) of uterus (ut), uterine tube (tu), ovary (ov) and vagina (vag); (L) longitudinal section through the uterine tube, fi: fimbriae, amp: ampulla; (M, P) fimbriae; (N, Q) ampulla; (O, R) isthmus of the uterine tube. Hematoxylin-eosin staining.

The ovaries were oval, slightly flattened, and presented the following measurements in adult females: a length of 2–2.5 cm, a width of 0.7–1 cm and a height of 0.4–0.5 cm. They were located caudal to the kidneys and oriented in a horizontal plane. A dense proper ligament of the ovary connected the medial pole to the base of the uterus (Fig. 5). The ovary was covered by a tunica albuginea of dense connective tissue and a simple cuboidal epithelium. The ovarian parenchyma consisted of the outer cortex and a thin central medulla. The cortical zone contained different stages of developing or degenerating follicles, and corpora lutea surrounded by irregularly arranged connective tissue (Figs. 6A–6D). Occasionally, agglomerations of cells with ovoid or spherical nuclei with granulated chromatin could be observed in the ovarian tissue. Most of those cord-like structures were solid, but in a few, a central lumen was beginning to canalize (Figs. 6G–6I).

### Uterine tubes

The uterine tubes were thin convoluted ducts, extending in the mesosalpinx from the ovaries to the fundic portion of the uterus (Figs. 6J, 6K). The distal portion of the uterine tube, the infundibulum, opened in the peritoneal cavity and surrounded the lateral pole of the ovary with fingerlike projections, the fimbriae (Figs. 6M, 6P). The infundibulum then continued in the ampulla with irregularly branching longitudinal mucosal folds, a simple cuboidal epithelium, and a thin muscular wall (Figs. 6N, 6Q). Gradually, the lumen and the number of longitudinal folds decreased, while the width of the muscular wall of the oviduct increased. The terminal part, the isthmus, penetrated the uterine wall and opened in the fundic portion of the uterus (Figs. 6O, 6R).

### Uterus

**Figure 7:**
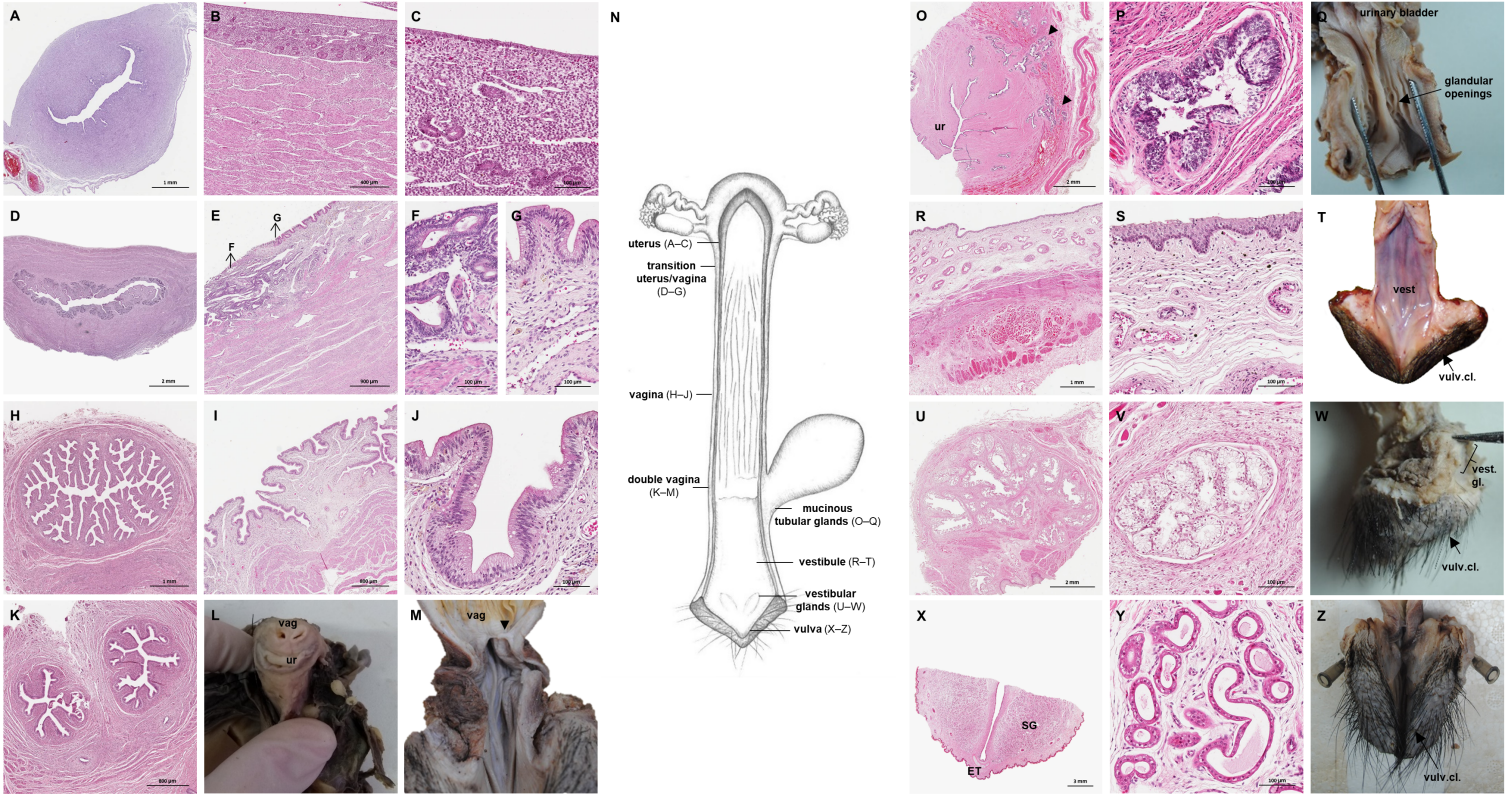
Macroscopic and microscopic aspects of the uterus, vagina, vaginal vestibule and glandular structures in the caudal genital organs of the adult and young female giant anteater. (A) Cross-section through the uterus of a young giant anteater; (B, C) endometrium and myometrium of an adult giant anteater in detail; (D) cross-section through the area of transition between uterus and vagina of a young giant anteater and (E) longitudinal section through the area of transition between uterus and vagina of an adult giant anteater, arrows indicating (F) section with uterine characteristics and (G) section with vaginal characteristics; (H–J) cross-section through the middle portion of the vagina and (K) photomicrograph and (L) photograph of a cross-section through the caudal vagina of a young giant anteater, displaying two lumina, vag: vagina, ur: urethra; (M) caudal vagina of an adult giant anteater, dorsal view, longitudinally cut, vag: vagina, arrowhead: septum separating the two vaginal lumina; (N) schematic drawing of the genital organs, dorsal view, longitudinally cut; (O, P) cross-section through the urethral wall near the opening into the vestibule of an adult giant anteater; (O) ur: urethral lumen, arrowhead: mucinous tubular glands; (P) mucinous tubular glands in detail; (Q) urethra of an adult giant anteater, longitudinally cut, arrow indicating location of the glandular structures; (R, S) vaginal vestibule and (T) caudal segment of the genital organs of an adult giant anteater, dorsal view, longitudinally cut, vest. : vestibule, vulv.cl.: vulvar cleft; (U, V) cross-section through the vestibular gland and (W) lateral view of the caudal segment of the genital organs of an adult giant anteater, vulv.cl: vulvar cleft, vest.gl.: vestibular glands dorsal to vulvar cleft; (X) cross-section through the vulva of a young giant anteater, ET: erectile tissue, SG: sudoriferous glands and (Y) agglomeration of sudoriferous glands; (Z) dorsal view of the vulvar cleft of an adult giant anteater. Hematoxylin-eosin staining.

The uterus was a simple pear-shaped organ with a thick muscular wall and was dorsoventrally flattened (Fig. 5). In adult animals, the uterine body measured a length of 3–5 cm, a width of 2.5–3 cm, and a height of 1.5–2 cm. The endometrium was lined by a simple cuboidal to columnar epithelium. Uterine glands of a simple tubular type opened on the endometrial surface and the submucosa was composed of connective tissue surrounding the uterine glands. The characteristics of the uterine glands and thickness of the endometrium varied with the oestrous cycle of the female. The myometrium consisted of bundles of smooth muscle (Figs. 7A–7C). The fundic portion of the uterus was covered by serosa, and received on each side the openings of the uterine tubes. The lumen of the uterus continued directly into the vaginal canal (Figs. 7D–7G). In two females with an involuting uterus, however, a constriction at the base of the uterus was observed.

### Vaginal canal

In adult animals, the vaginal canal extended approximately 6–10 cm caudally, presented a wide lumen and a thin wall with longitudinal mucosal folds, lined by a simple columnar epithelium (Figs. 7H–7J). The caudal portion of the vagina had two lumina that were separated by a longitudinal septum (Figs. 7K–7M). The thickness of the septum varied among the specimens: in young and occasionally in adult animals, the septum was thick, whereas it was reduced to a thin membrane in most adult specimens. The double vagina opened with two apertures into the vaginal vestibule, directly dorsal to the opening of the urethra.

### Persisting Wolffian ducts

**Figure 8:**
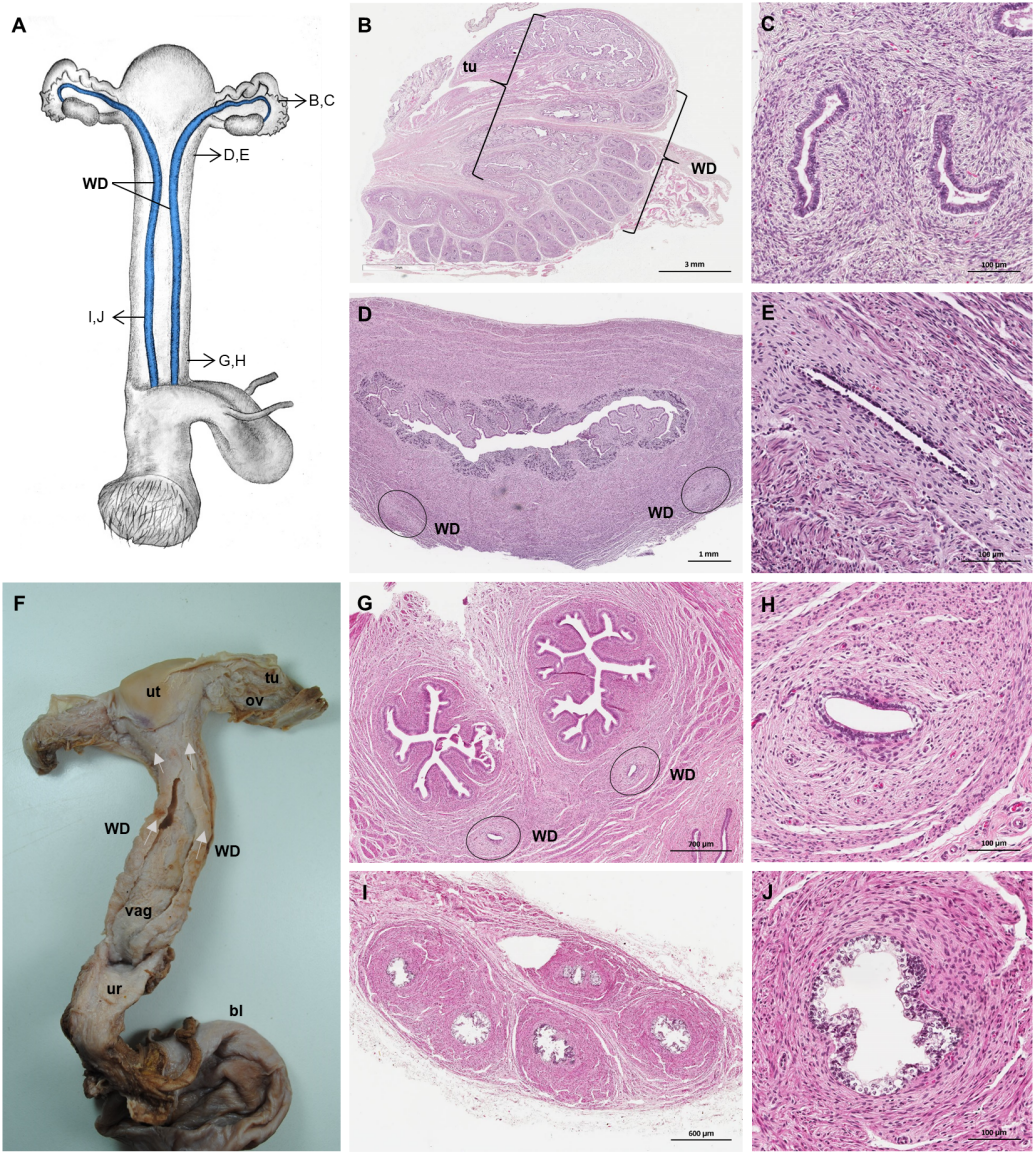
Macroscopic and microscopic aspects of the persisting Wolffian duct structures in the adult and young female giant anteater. (A) Schematic drawing of the genital organs, ventral view, in blue: persisting Wolffian ducts (WD), arrows: indicating regions for histological sections (B, D, G, I); (B) longitudinal section through the uterine tube in an adult giant anteater (tu) with accompanying Wolffian duct (WD); (C) Wolffian duct in B in detail; (D) cross-section through the area of transition from uterus to vagina in a young giant anteater, encircled: Wolffian ducts (WD) passing through the muscular wall; (E) Wolffian duct in D in detail; (G) cross-section at the height of the double vagina in a young giant anteater, encircled: Wolffian ducts (WD) passing through the vaginal wall; (H) Wolffian duct in G in detail; (I) cross-section through the Wolffian duct in an adult giant anteater; (J) Wolffian duct in I in detail; (F) photograph of the genital organs with dissected Wolffian ducts in an adult giant anteater, ventral view, bl: bladder; ur: urethra, vag: vagina; ut: uterus; ov: ovary; tu: uterine tube; WD: Wolffian duct; arrows: indicating the course of the Wolffian ducts.

Well-differentiated remnants of the Wolffian (or mesonephric) ducts were observed in all examined female giant anteaters (Fig. 8). Two ducts with a thin muscular wall and a central lumen lined by a simple cuboidal epithelium originated in the ventral vaginal wall at the height of the vaginal opening into the vestibule. The ducts passed in a cranial direction through the ventral vaginal and uterine wall (Figs. 8D–8H). Finally, the diameter of the ducts decreased, and each duct extended highly coiled alongside the ipsilateral uterine tube until the hilus at the lateral pole of the ovary (Figs. 8B, 8C). There, a highly ramified network of small ducts formed a rete ovarii with a simple cuboidal epithelium (Fig. 6E).

### Vestibule and Vulva

In the vestibule, the vaginal columnar epithelium changed into a stratified squamous epithelium resting on highly vascularized connective tissue with an extensive venous plexus (Figs. 7R–7T). The urethra opened on the floor of the vestibule. Mucinous tubular glands with columnar epithelium could be found in the urethral wall near the opening into the vestibule and corresponded to a male prostate gland (Figs. 7O–7Q). Furthermore, a compound of tubuloalveolar glands similar to male bulbourethral glands was located in the lateral wall of the vestibule. The acini of those vestibular glands were lined by a simple columnar epithelium (Figs. 7U–7W). The vestibule opened to the exterior through a vertical vulvar cleft framed by two labia. At the ventral commissure of the vulva, a half-moon shaped erectile tissue could be observed, flanked laterally on each side by an agglomeration of sudoriferous glands with numerous secretory tubules with simple cuboidal epithelium (Figs. 7X–7Z). The vulva was situated directly ventral to the anus and the conical shape resembled the penis of the male animal. The only external difference to the male copulatory organ was a longitudinal cleft of 3–5 cm opening into the vestibule. In adult animals, the length of the vestibule from the ventral vulvar commissure to the vaginal opening was 7–10 cm.

## Discussion

Both male and female giant anteaters possess unique morphological characteristics of the reproductive tract, adding to the peculiar features that single out the species among other mammals.

### Male genital organs

In male giant anteaters, a notable feature is the intraabdominal position of the testes, a shared characteristic among all Xenarthrans. In anteaters (Kaudern, 1914; Bartmann, Beyer & Wißdorf, 1991; Rossi et al., 2012) and sloths (Grassé, 1955; Barretto, Amorim & Falcão, 2013), the testes are found caudomedially to the kidneys and joined to each other. In contrast, in armadillos, the testes occupy a more caudal and inguinal position, some authors mentioning the presence of a short peritoneal protrusion into the inguinal canal (Rapp, 1852; Kaudern, 1914; Newfang, 1947). Intraabdominal testes occur also in most afrotherian species, Eulipotyphla, and Pholidota (Kleisner, Ivell & Flegr, 2010; Lovegrove, 2014). It is still controversial whether the undescended testes position is an ancestral condition or a derived characteristic in placental mammals. Some authors argue that a descended scrotal position of the testes evolved early in mammalian evolution, and was independently lost in several lineages (Werdelin & Nilsonne, 1999; Sharma et al., 2018; Chai et al., 2021). In contrast, Kleisner, Ivell & Flegr (2010) and Lovegrove (2014) suggest that abdominal testes represent the ancestral condition in (eutherian) mammals. However, the cited studies are either based on outdated phylogenetic data (Werdelin & Nilsonne, 1999) or assumptions concerning the location of testes in Xenarthrans that are either incorrect (testicular descent, Sharma et al., 2018) or imprecise, not considering the differences in the position of the testes in anteaters and sloths compared with armadillos (as described in: Kaudern, 1914; Rossi et al., 2012; Barretto, Amorim & Falcão, 2013).

Some authors (Werdelin & Nilsonne, 1999; Rodrigues et al., 2008) suggest that the internal location of the testes in Xenarthrans is linked to their low body temperature (Aguilar & Superina, 2015: around 30 °C to 35 °C), which could ensure functional spermatogenesis despite the internal location of the gonads. Cryptorchid testes in other mammalian species are usually prone to defects in sperm production (Setchell, 2006).

In addition to the absence of a scrotum, the small and conical penis of the giant anteater contributes to the difficulty of differentiating male and female individuals by simple inspection. As stated above, the penis resembles the female vulva in size and shape, but males can be identified by a small urethral orifice at the free extremity of the penis, this contrasting with a longitudinal vulvar cleft in the female. Likewise, the external genital organs can be easily confounded in male and female sloths (Favoretto et al., 2015). For giant anteaters, Bartmann, Beyer & Wißdorf (1991) mentioned a scrotum-like structure directly ventral to the anus. In the present study, however, this structure proved to be merely the slightly concave dorsal face of the penis. Due to the small size of the penis and limited erectile tissue and function, Bartmann, Beyer & Wißdorf (1991) expect only a shallow penetration during copulation of giant anteaters. Data of this study suggest that the penis does not penetrate the vagina due to a vestibular length of 7–10 cm in the female and a penis length of only 5–6 cm. The extensive sudoriferous glands observed in the distal segment of the penis might play a role in intraspecific chemical communication.

Similar to other mammalian species (König & Liebich, 2014), the giant anteater possesses three accessory sexual glands that open into the urethra and contribute with secretion to the seminal plasma: the prostate, vesicular and bulbourethral glands. The same was observed in other xenarthran species: the silky anteater (*Cyclopes didactylus*), the southern tamandua (Kaudern, 1914; Rossi et al., 2012), and the nine-banded armadillo (Glover, 1963; Cardoso et al., 1985). In the two-toed sloth (*Choloepus hoffmanni*) and Bradypus spp., Wislocki (1928) and Grassé (1955) state that the bulbourethral glands were missing. In the present study, the distance from the free extremity of the penis to the seminal colliculus measured around nine cm in adult giant anteaters, and might serve as a landmark when executing semen collection procedures, such as electroejaculation, transrectal massage or urethral catheterization.

The well-differentiated remnant of the Müllerian ducts described in this study has been previously identified in giant anteaters as *uterovagina masculina* by Kaudern (1914) and Bartmann, Beyer & Wißdorf (1991). The observations by Kaudern (1914), however, are based on an incomplete reproductive tract of one specimen and Bartmann, Beyer & Wißdorf (1991) do not provide any macroscopic or microscopic details. The structure is most likely the result of some aberration during fetal sexual differentiation in male individuals.

Sexual differentiation in mammals is a highly complex process and various developmental aberrations can cause retention of vestigial genital structures of the opposite sex. Both genetically male and genetically female embryos possess a pair of bipotential gonads and two pairs of genital ducts, the Wolffian and the Müllerian ducts, which are precursors of male and female genital organs, respectively (MacLaughlin, Teixeira & Donahoe, 2001; Ohnesorg, Vilain & Sinclair, 2014). Sex determination begins when the bipotential gonads differentiate into either testes or ovaries: in genetic males, testes differentiation starts when a gene on the Y chromosome, the SRY gene, is expressed in the embryonic gonad and initiates the development of Sertoli and Leydig cells (Albrecht & Eicher, 2001; Kanai et al., 2005; She & Yang, 2016). Shortly after the onset of SRY expression, SOX9 is upregulated (Ohnesorg, Vilain & Sinclair, 2014; Stévant, Papaioannou & Nef, 2018) and can substitute for all functions of SRY (Kanai et al., 2005). Leydig cells in the fetal testis secrete testosterone, which causes the differentiation of the male genital duct and urogenital sinus into the epididymis, deferent duct, and accessory sexual glands (Dyche, 1979; MacLaughlin, Teixeira & Donahoe, 2001; Marker et al., 2003; Edwards, Moore & Guillette Jr, 2006; Welsh, Suzuki & Yamada, 2014). Furthermore, testosterone initiates the formation of the external male genitalia from the genital tubercle (MacLaughlin, Teixeira & Donahoe, 2001; Welsh, Suzuki & Yamada, 2014). The Sertoli cells in the fetal gonad produce the Anti-Müllerian Hormone (AMH: She & Yang, 2016), which binds to the Anti-Müllerian Hormone receptor type 2 (AMHR2) on the surface of Müllerian duct mesenchymal cells, thus causing regression of the female genital duct (Dyche, 1979; Stévant, Papaioannou & Nef, 2018).

Rudimentary cystic or tubular remnants of the Müllerian ducts have been observed sporadically in humans (Drake, Vogl & Mitchell, 2008), several domestic species (Swoboda, 1929), and even some Xenarthrans (Kaudern, 1914). Better differentiated developmental remnants of the Müllerian ducts with distinct uterine and vaginal characteristics are linked to the Persisting Müllerian Duct Syndrome (PMDS) that occurs in otherwise phenotypically normal male individuals (XY), and is mostly connected to a mutation in AMH, AMHR2 or downstream signaling pathways: the PMDS sporadically affects humans (Josso et al., 2005; Picard et al., 2017), interestingly 85% of the European bison (*Bison bonasus*; Panasiewicz et al., 2015), and is also frequently observed in the miniature schnauzer (Lyle, 2007; Wu et al., 2009; Vegter et al., 2010; Meyers-Wallen, 2012). Only very few species are known where Müllerian duct structures persist in all male individuals: some treeshrew species (Tupaia spp.; Alcalá & Conaway, 1968) and beavers (Castor spp.; Conaway, 1958; Doboszynska & Zurowski, 1981) are mentioned in literature. Similarly, in the present study, the Müllerian duct remnants were observed in all male giant anteaters and constituted the normal morphological condition in this species. In contrast to the beaver, where highly variable interindividual characteristics of those embryonic structures were observed (Conaway, 1958), the morphology of the Müllerian duct remnants differed little between male giant anteaters. This was even more remarkable as the remnants were not just a miniature version of the female genital organs but presented very distinct characteristics: macroscopically, the tube-like uterine and bilobed vesicular vaginal structure in the male did not resemble the female simple uterus with adjacent straight vaginal canal.

In literature, a confusing multitude of terms has been applied to remnants of the Müllerian ducts, ranging from *utriculus masculinus* (Swoboda, 1929; Drake, Vogl & Mitchell, 2008) to *uterus masculinus* (Kaudern, 1914; Conaway, 1958) or *vagina masculina* (Alcalá & Conaway, 1968). None of the aforementioned terms correctly describe the structure observed in the present study in male giant anteaters, which presents characteristics of uterus and vagina as well as uterine tubes.

The presence of the Müllerian duct remnant does not affect fertility in males, as it occurs in all male individuals of the species. The structure even has secretory functions and the yellowish secretion in its lumen, described in this study, presumably contributes to the seminal plasma. In electroejaculation procedures, ejaculate fractions with the same aspect have been collected in giant anteaters (personal observation, unpublished data). In other species, pathologies of Müllerian duct remnants have been reported, ranging from cystic degeneration (Atilola & Pennock, 1986; Ritchey et al., 1988; Kato et al., 2002) or neoplasia (Vignoli et al., 2020) to male pyometra (Wu et al., 2009). It is important to bear in mind these potential pathologies in reproductive medicine of the giant anteater. The etiology of the persistence of Müllerian duct structures in males is currently unexplained. Future research should therefore examine possible mutations of the AMH or AMHR2 genes or other involved factors.

### Female genital organs

Unlike most other placental mammalian species, the female giant anteater possesses a simple uterus. Common uterine morphologies among placentalia are a bicornuate or double uterus (Kobayashi & Behringer, 2003). A simple uterus is rare, mainly occurring in primates (Kobayashi & Behringer, 2003; Hayssen & Orr, 2017), but has been described in other xenarthran species, like the southern tamandua (Rossi et al., 2011), sloths, and armadillos (Cetica, Aldana Marcos & Merani, 2005; Favoretto et al., 2015). The uterus of the female giant anteater continues directly in a long and thin-walled vaginal tube without forming a distinct cervical segment. In this study, however, in female specimens with an involuting uterus, a constriction at the base of the uterus was identified, which might serve as a microbiological and mechanical barrier during pregnancy. Similarly, Schauerte & Osmann (2012) observed a zone of more densely folded mucosa at the transition of the uterine body to the vagina in a female deceased intrapartum.

The vagina in the giant anteater, in contrast to the stratified squamous vaginal epithelium commonly observed in mammalian species (Liebich, 2010), is lined by columnar epithelium. It therefore resembles the columnar epithelium of the endocervix described in other mammals (Liebich, 2010). Yet, the anatomical region between the uterus and opening of the urethra corresponds to a vagina. Furthermore, the thin-walled tube does not show gross morphological similarities to the cervix of other mammalian species, such as a thick muscular wall and the capacity to function as a barrier (Liebich, 2010). Interestingly, the caudal part of the vagina in the female giant anteater displays two lumina. During organogenesis of the reproductive tract, the Müllerian ducts apparently fuse in the middle segment, forming a simple uterus and the cranial part of the single vaginal tube. Fusion is incomplete, however, in cranial and caudal segments of the Müllerian ducts, resulting cranially in paired uterine tubes, and caudally in a double vagina. Differences in the extent of fusion of the Müllerian ducts have been observed throughout mammalian species: marsupials with a double uterus and vagina at one extreme and primates with a simple uterus and vagina at the other extreme (Kobayashi & Behringer, 2003). Female giant anteaters with a simple uterus but double caudal vagina occupy a singular position here. Nevertheless, these findings match the theory of bidirectional fusion of the Müllerian ducts during formation of the female reproductive tract (Acién, Acién & Sánchez-Ferrer, 2009), according to which the fusion of the Müllerian ducts starts at the uterine isthmus and then continues in a cranial and caudal direction.

In the literature on the reproductive morphology of Xenarthrans, similar characteristics as revealed here, have been observed. However, while authors agree on the presence of a simple uterus, various terms have been employed to describe the tubular structure that was called the vagina in the present study: vagina (Owen, 1868: *Dasypus peba*; Wislocki, 1928: *Bradypus griseus griseus*, *Choloepus hoffmanni peters*; Schauerte & Osmann, 2012: *Myrmecophaga tridactyla*), uterovaginal canal (Rossi et al., 2011: *Tamandua tetradactyla*), a segment of the uterus (Rapp, 1852: edentates; Newfang, 1947: *Dasypus novemcinctus*; Favoretto et al., 2015: *Bradypus variegatus*) or cervix (Cetica, Aldana Marcos & Merani, 2005: several armadillo species). The double lumina observed in the present study in the caudal segment of the tubular structure denominated vagina here were observed as well in the southern tamandua and sloths (Rapp, 1852; Rossi et al., 2011), however, not in armadillos (Newfang, 1947; Cetica, Aldana Marcos & Merani, 2005).

In the ovaries of some giant anteaters, cord-like structures composed of cells with ovoid or spherical nuclei and occasionally the beginning of canalisation of a central lumen were observed. These structures resemble the seminiferous cords of fetal mammalian testes, which are characterized as solid cords composed of sustentacular Sertoli cells and primordial germ cells, which later on canalize to form the seminiferous tubules in the testes of adult animals (Dyce, Sack & Wensing, 2010).

Similar to male giant anteaters, which retain well-developed remnants of the female genital ducts, male morphological characteristics were described in the genital organs of all female specimens in this study. Apart from the seminiferous cord-like structures in the ovaries, glandular structures that are homologs of the male prostate and bulbourethral glands are well-developed, and coiled Wolffian ducts run from the ovaries along the uterus and vagina, possibly due to some species-specific peculiarities during female sexual differentiation. While male sexual differentiation in placental mammals is induced by the Y chromosome-linked SRY-gene leading to the differentiation of the bipotential gonad into a testis, female sexual differentiation occurs in the absence of the SRY gene and under influence of X-linked genes, beginning with the differentiation of the bipotential gonad into ovaries (Mackay, 2000; Gardiner & Swain, 2014). Normally, in the absence of testosterone, the Wolffian ducts degenerate and, during further sexual differentiation, the Müllerian ducts develop into uterine tubes, the uterus, and the cranial portion of the vagina, while the female external genitalia develop autonomously without hormonal stimuli from the genital tubercle (MacLaughlin, Teixeira & Donahoe, 2001).

As detailed above, the process that leads to the development of genital organs is distinct for male and female mammals. Accordingly, the etiology of persisting genital duct structures of the opposite sex is not identical in male and female giant anteaters. The formation of supposedly seminiferous cord-like structures in the ovaries might be the result of some SRY downstream mutation in females. Female giant anteaters are not carriers of the SRY gene itself (Takami et al., 1998; Barragán-Ruiz et al., 2021), but other factors like SOX9 might be upregulated and lead to the formation of cords with Sertoli-like cells in the ovaries. Testis or medullary cords were also observed in fetal and adult ovaries of some armadillo species (Newfang, 1947; Enders, 1960; Cetica, Aldana Marcos & Merani, 2005). Furthermore, Cetica, Aldana Marcos & Merani (2005) identified rudimentary Wolffian duct remnants, such as epoophoron and a rete ovarii in female armadillos. Small remnants of the degenerating Wolffian ducts may also persist in humans (Hannema & Hughes, 2007). The presence of continuous and differentiated Wolffian ducts in all female giant anteaters, however, is an extraordinary finding, since development of those ducts requires an androgen source during sexual differentiation (Dyche, 1979; MacLaughlin, Teixeira & Donahoe, 2001; Welsh et al., 2009). Glandular structures observed in female giant anteaters, corresponding to male prostate and bulbourethral glands, have been mentioned in other mammalian species as well: homologs to the male prostate gland at the base of the bladder are denominated Skene gland (Zaviacic et al., 2000; Santos & Taboga, 2006), and the paired glands opening laterally into the vaginal vestibule are called major vestibular glands (Dyce, Sack & Wensing, 2010; König et al., 2014). The homologs in male mammals are derived from the urogenital sinus and, similar to the Wolffian ducts, differentiate under the influence of androgens (Marker et al., 2003). Whether the ovarian tissue with seminiferous cord-like structures in giant anteaters could function as a source of androgens during female sexual differentiation, and thus induce differentiation of the Wolffian ducts and glandular structures, needs to be clarified.

Similar to the Müllerian duct remnants in male giant anteaters, the persisting Wolffian ducts and the above-described glandular structures might have secretory functions in the female reproductive tract. Still, those structures could also give rise to pathological conditions mentioned in other mammalian species. Neoplasia, cystic degeneration or abscesses have been observed in remnants of Wolffian ducts as well as Skene glands and major vestibular glands in women (Blackwell & McElin, 1955; Copeland et al., 1986; Dodson et al., 1994; Omole, Simmons & Hacker, 2003).

There are only a few mammalian species where females develop male characteristics in genital organs. Female Iberian moles (*Talpa occidentalis*) possess bilateral ovotestes with both ovarian and testicular tissue, rudimentary epididymides, and a peniform clitoris but otherwise normally differentiated female genital organs (Jiménez et al., 1993; Zurita et al., 2003; Barrionuevo et al., 2004). Those females lack the SRY gene and Jiménez et al. (1993) suppose that a mutation in a non-Y-linked gene involved in the testis-determining pathway leads to the formation of testicular tissue in female gonads. In these ovotestes, Leydig cells produce testosterone and cause the differentiation of rudimentary epididymides (Jiménez et al., 1993), but Sertoli cells do not express AMH and therefore the Müllerian ducts develop into normal female genital organs (Zurita et al., 2003). Another example are female spotted hyaenas (*Crocuta crocuta*): internal genital organs are normally differentiated, but external genitals resemble a penis with false scrotum (Neaves, Griffin & Wilson, 1980; Lindeque & Skinner, 1982). In this case, both androgenic and non-androgenic mechanisms are involved: placental conversion of maternal ovarian androstenedione to testosterone and low serum concentrations of sex hormone-binding globulins, resulting in higher concentrations of free dihydrotestosterone (Glickman et al., 1992; Conley et al., 2020). The cases of Iberian moles and spotted hyaenas demonstrate that both androgens and other factors might be involved in the formation of male characteristics in the genital organs of females.

## Conclusions

The giant anteater shows unique characteristics of the reproductive tract. Some features such as intraabdominal testes or a simple uterus are shared among xenarthran species. The persistence of well-developed genital duct structures of the opposite sex in both male and female giant anteaters, however, singles them out among other xenarthrans as well as mammalian species in general. Further investigations on the etiology and possible secretory functions of these remnants of the Müllerian and Wolffian ducts can help to better understand the reproductive peculiarities of this threatened species, and might also shed light on sexual differentiation in placental mammals in general.

## Additional Information and Declarations

### Data Availability

The following information was supplied regarding data availability: The raw data on identification and storage of the studied specimens is available in the Supplementary File.
